# Inferring Conformational State of Myosin Motor in an Atomic Force Microscopy Image *via* Flexible Fitting Molecular Simulations

**DOI:** 10.3389/fmolb.2022.882989

**Published:** 2022-04-29

**Authors:** Sotaro Fuchigami, Shoji Takada

**Affiliations:** Department of Biophysics, Graduate School of Science, Kyoto University, Kyoto, Japan

**Keywords:** atomic force microscopy, myosin V, actin filament, flexible fitting, coarse-grained molecular simulation, CafeMol

## Abstract

High-speed atomic force microscopy (HS-AFM) is a powerful technique to image the structural dynamics of biomolecules. We can obtain atomic-resolution structural information from the measured AFM image by superimposing a structural model on the image. We previously developed a flexible fitting molecular dynamics (MD) simulation method that allows for modest conformational changes when superimposed on an AFM image. In this study, for a molecular motor, myosin V (which changes its chemical state), we examined whether the conformationally distinct state in each HS-AFM image could be inferred *via* flexible fitting MD simulation. We first built models of myosin V bound to the actin filament in two conformational states, the “down-up” and “down-down” states. Then, for the previously obtained HS-AFM image of myosin bound to the actin filament, we performed flexible-fitting MD simulations using the two states. By comparing the fitting results, we inferred the conformational and chemical states from the AFM image.

## 1 Introduction

High-speed atomic force microscopy (HS-AFM) is a powerful tool for observing the structural dynamics of biomolecules under near-physiological conditions at a single-molecule resolution (Ando et al., 2001; Kodera et al., 2010; Uchihashi et al., 2011; Casuso et al., 2012; Ando et al., 2014; Uchihashi and Scheuring, 2018; Ando, 2019). Despite its unique advantages, AFM directly measures only the molecular surface of the specimen. To obtain three-dimensional structural information about the molecule, we need to model a structure that is consistent with the AFM image. When such a consistent structural model is available beforehand, the optimal position and orientation of the model in the AFM image can be determined only by translating and rotating a given model, that is, using a rigid-body fitting method (Scheuring et al., 2005; Scheuring et al., 2007; Asakawa et al., 2011; Trinh et al., 2012; Chaves et al., 2013; Dasgupta et al., 2020; Niina et al., 2021). However, rigid-body fitting cannot be applied to the cases wherein molecules change their shapes from the available structure models. Recently, to overcome this problem, we developed a flexible fitting molecular dynamics (MD) method to find structures compatible to the AFM image and applied the method to two HS-AFM images (Niina et al., 2020a). In the flexible fitting MD simulation, we added a bias potential that quantifies the compatibility with the AFM image to the standard structure-based potential. As structure-based potential refers to a given reference structure, flexible fitting MD works better when a modest structural change from the reference is sufficient to fit with the AFM image. However, it does not work well when the AFM image represents a distinct conformational state from the reference structure.

Various proteins fulfill their functions through transitioning into distinct conformational states upon ligand binding, chemical reactions, and release (Fuchigami et al., 2011). A representative example is an ATP-dependent motor protein that converts chemical energy into mechanical force *via* the ATP hydrolysis cycle. When HS-AFM is used to observe the functional motions of motor proteins, the chemical states of these proteins change over time. However, HS-AFM cannot directly detect the bound nucleotides. Thus, important questions arise if we can infer the chemical state of proteins in each HS-AFM frame, which we address in this report using myosin V as a test case.

**FIGURE 1:**
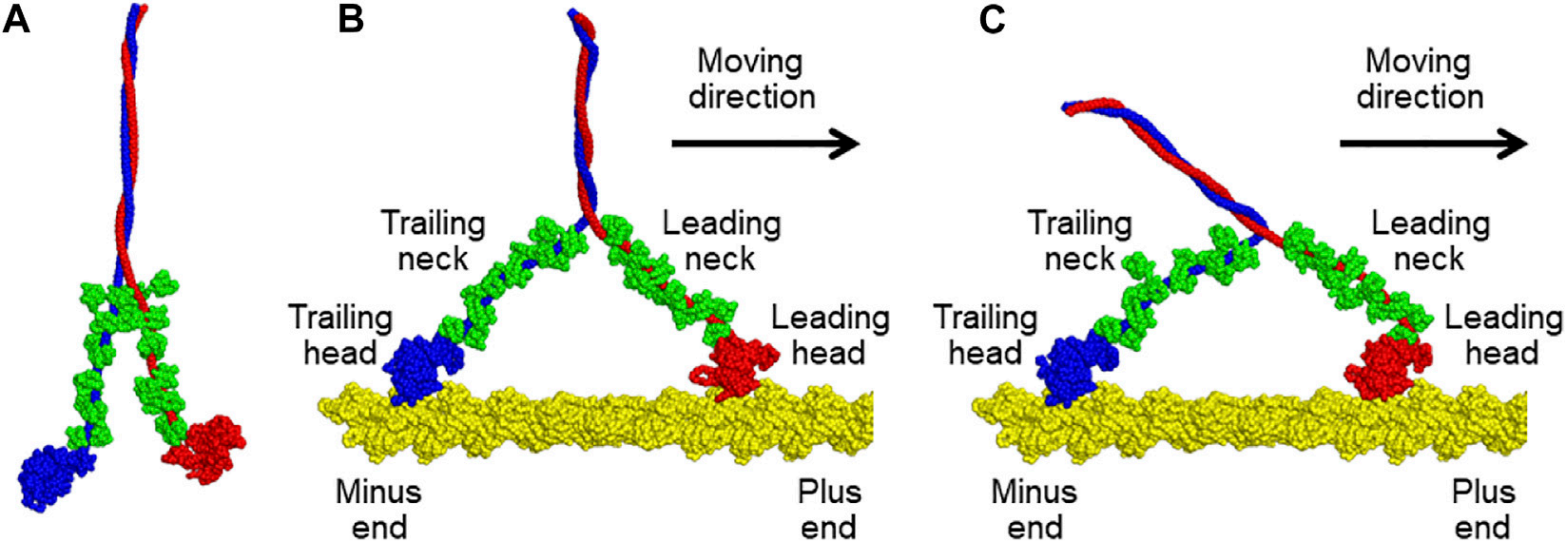
**(A)** Molecular model of a tail-truncated myosin V. **(B,C)** Molecular models of the complexes of an actin filament with myosin V in **(B)** the down-up state and **(C)** down-down state. Two heavy chains of myosin V are shown in blue and red. Twelve light chains of myosin V in compact or extended conformations are colored in green. The actin filament is indicated in yellow. All structures were drawn using PyMOL version 1.8.4.0 (http://www.pymol.org/).

Myosin V is one of the best-characterized motor proteins that walks unidirectionally along the actin filament, driven by ATP hydrolysis free energy, to transport cargo *via* the cytoskeleton (Sellers and Veigel, 2006; Hammer and Sellers, 2011). Myosin V takes a homodimeric form connected *via* the coiled-coil region (top side in Figure 1A). Each monomer contains a motor domain, also called the head domain (the bottom globules in Figure 1A), and a neck domain that connects the head and the coiled coil. Additionally, the neck domain binds six calmodulins (green in Figure 1A). The two heads of myosin V alternatively change their lever-arm conformations between the upward and downward states coupled to the ATP hydrolysis cycle, leading to walking on an actin filament. Kodera et al. observed such movements of myosin V using HS-AFM in real-time (Kodera et al., 2010). However, the spatial resolution of the obtained AFM images is insufficient to reveal the details of the molecular structure.

In this brief report, we focus on the structure of myosin V bound to an actin filament visualized *via* HS-AFM (Kodera et al., 2010), and aim to generate a structure that fits well with the AFM image. Notably, the AFM image does not directly detect the bound nucleotide; thus, the chemical state of myosin in each time frame is unknown *a priori*. We first modelled tail-truncated myosin V in two conformational states, the “down-up” and “down-down” states, as well as the structure of the actin filament. We then performed flexible-fitting MD simulations of the actin-myosin complex, which depends on the chemical state of myosin. Using cosine similarity between an experimental AFM image and a pseudo-AFM image of the optimal structure, we estimated the conformational state of myosin V.

## 2 Method

### 2.1 Molecular Models of Myosin V and the Actin Filament

We constructed a molecular model of a tail-truncated myosin V and an actin 31-mer filament *via* homology modelling using Modeller version 9.25 (Šali and Blundell, 1993). Tail-truncated myosin V is composed of two heavy chains (1140 residues) and 12 light chains of calmodulins (148 residues). For the modelling of myosin V, we first divided it into 15 regions—two motor domains of the heavy chain, twelve complexes of IQ motif of the heavy chain and light chain, and one coiled-coil domain formed by two heavy chains. The two motor domains were modelled in two different conformations of the lever-arm down and lever-arm up states. As the template for homology modelling, the nucleotide-free structure of myosin V (PDB ID: 1OE9, chain ID: A) was used for the lever-arm down conformation, and two nucleotide-bound structures of myosin Vc (PDB ID: 5HMP, chain ID: B) and myosin 11 (PDB ID: 1BR1, chain ID: A) were used for the lever-arm up conformation. The light chain bound to the IQ motif can adopt compact and extended conformations, and the light chain conformation can be inferred from the sequence of the bound IQ motif based on the study of homologous complexes (Terrak et al., 2005) as follows: eight light chains bound to IQ1, IQ2, IQ4, and IQ5, and four light chains bound to IQ3 and IQ6 are in compact and extended conformations, respectively. The compact complex was modelled using the structures of the homologous complex (PDB ID: 1N2D, chain ID: A and C) and light chain (PDB ID: 6C1H, chain ID: R). The extended complex was modelled using the structures of the homologous complexes (PDB ID: 1M46, chain ID: A and B; PDB ID: 1N2D, chain ID: A and C) and light chain (PDB ID: 6C1H, chain ID: R). The main chain of the coiled-coil domain was generated using the CCFold web server (Guzenko and Strelkov, 2017), and its side-chain conformation was predicted using SCWRL (Krivov et al., 2009). Finally, the obtained structures of all regions were connected to construct the overall structure of myosin V, as shown in Figure 1A. On the other hand, the actin 31-mer filament was created by starting with an actin 5-mer filament (PDB ID: 6C1H, chain ID: A, B, C, D, and E) and elongating using another actin 5-mer filament by overlapping the terminal actin monomers.

Next, we generated two different molecular models of the actin-myosin complex in which both heads of myosin V were bound to the actin filament. In the two models, the trailing head of myosin V was in the same lever-arm down conformation, but its leading head differed in the lever-arm conformation, up or down, as shown in Figures 1B,C. The conformational states of the models are called the down-up and down-down states based on the lever-arm conformation. First, a molecular model of the actin-myosin complex in the down-up state was generated as follows. The generated molecular model of myosin V was moved to a position where the helix-turn-helix (HTH) motif of the leading head interacts with the actin filament. Then, a coarse-grained (CG)-MD simulation was performed with the structure-based potential so that the structure in the down-up state is the most stable and a harmonic potential to the HTH motif of the trailing head such that the HTH motif interacts with the actin filament. By gradually increasing the strength of the potential in five steps, we succeeded in obtaining a myosin V structure in the down-up state that properly bound to actin filaments (Figure 1B). To generate a molecular model of the actin-myosin complex in the down-down state, a further CG-MD simulation was performed by switching the structure-based potential so that the structure of myosin V in the down-down state was the most stable. Through this simulation, we successfully obtained myosin structure in the down-down state (Figure 1C).

### 2.2 Atomic Force Microscopy Image Transformation for Flexible Fitting Molecular Dynamics Simulation

In the AFM measurement, the surface of the AFM stage was not perfectly parallel to the *XY* plane but was often slightly tilted (less than 1°). To model the AFM stage in the flexible-fitting MD simulation, the use of a stage potential completely parallel to the *XY* plane is preferred. Therefore, we transformed the AFM image such that the stage surface was parallel to the *XY* plane. For AFM image transformation, we first estimated the stage surface from the experimental AFM image. The heights of pixels in the region considered to be the stage surface were extracted from the experimental AFM image using the Python library of libasd (Niina and Koide, 2021) and were fitted to a plane using the linear least squares method. The AFM image was then transformed such that the obtained plane was *z* = 0.

In this study, we used an AFM image of the tail-truncated myosin V bound to the actin filament with two heads with an imaging area of 150 × 75 nm^2^ and 80 × 40 pixels. This AFM image was transformed using 38 × 10 pixels in the upper right region (43rd–80th pixels in the *X* direction and 30th–39th pixels in the *Y* direction), which is supposed to be the AFM stage surface. The estimated surface was represented as z=0.00348x−0.00003y+7.21493 nm and was very close to, but not identical to, the *XY* plane.

### 2.3 Flexible Fitting Molecular Dynamics Simulation Using Coarse-Grained Model

In the flexible fitting MD simulation, we express the total potential energy function as $V_{\text{total}}=V_{\text{protein}}+V_{\text{stage}}+V_{\text{AFM}}$ , where $V_{\text{protein}}$ , $V_{\text{stage}}$ , and $V_{\text{AFM}}$ represent the force field potential for proteins, AFM stage potential, and bias potential that quantifies the incompatibility between the simulated structure and AFM image, respectively. In this study, we used a coarse-grained model of a protein (in which each amino acid residue was represented as a single particle) with structure-based AICG2+ potential (Li et al., 2014). To model the interaction between a protein and the AFM stage, a Lennard-Jones stage potential was added (Niina et al., 2020a). The AFM image-based bias potential (Niina et al., 2020a) is defined as $V_{\mathrm{AFM}}=\;k\left(1-c.s.\left(H^{\left(\mathrm{sim}\right)};H^{\left(\mathrm{ref}\right)}\right)\right)$ , where *k* is a parameter of the bias strength. The $c.s.\left(H^{\left(\text{sim}\right)};H^{\left(\text{ref}\right)}\right)$ is the cosine similarity between the pseudo-AFM image generated from a snapshot in the simulation *via* a smoothed method, $H^{\left(\text{sim}\right)}$ , and the reference AFM image, $H^{\left(\text{ref}\right)}$ , defined as

$$c.s.\left(H^{\left(\text{sim}\right)};H^{\left(\text{ref}\right)}\right)=\;\frac{\sum_{p\in \text{pixels}}H_{p}^{\left(\text{sim}\right)}H_{p}^{\left(\text{ref}\right)}}{\sqrt{\sum_{p\in \text{pixels}}{\left(H_{p}^{\left(\text{sim}\right)}\right)}^{2}}\sqrt{\sum_{p\in \text{pixels}}{\left(H_{p}^{\left(\text{ref}\right)}\right)}^{2}}}$$

$H^{\left(\text{sim}\right)}$ has three parameters, *σ*_*x*_, *σ*_*y*_, and *γ* (*σ*_*x*_ and *σ*_*y*_ specify the ranges in *x* and *y* directions and *γ* determines a degree of smoothing in *z* direction), which were set to 0.5, 0.5, and 0.1 nm, respectively. The AFM probe tip radius corresponding to *σ*_*x*_ = *σ*_*y*_ = 0.5 nm was ∼2.6 nm based on our previous work (Fuchigami et al., 2021); this value was in the range of the typical AFM probe tip radius (Ando et al., 2013).

All flexible fitting simulations of the actin-myosin complex in the down-up and down-down states were performed with CafeMol version 3.2.0 (Kenzaki et al., 2011) using Langevin dynamics at a temperature of 300 K for 3 × 10^6^ MD steps, with an MD step of 0.3 in CafeMol time units.

## 3 Results

### 3.1 Flexible Fitting Molecular Dynamics Simulation With the Down-Up State

**FIGURE 2:**
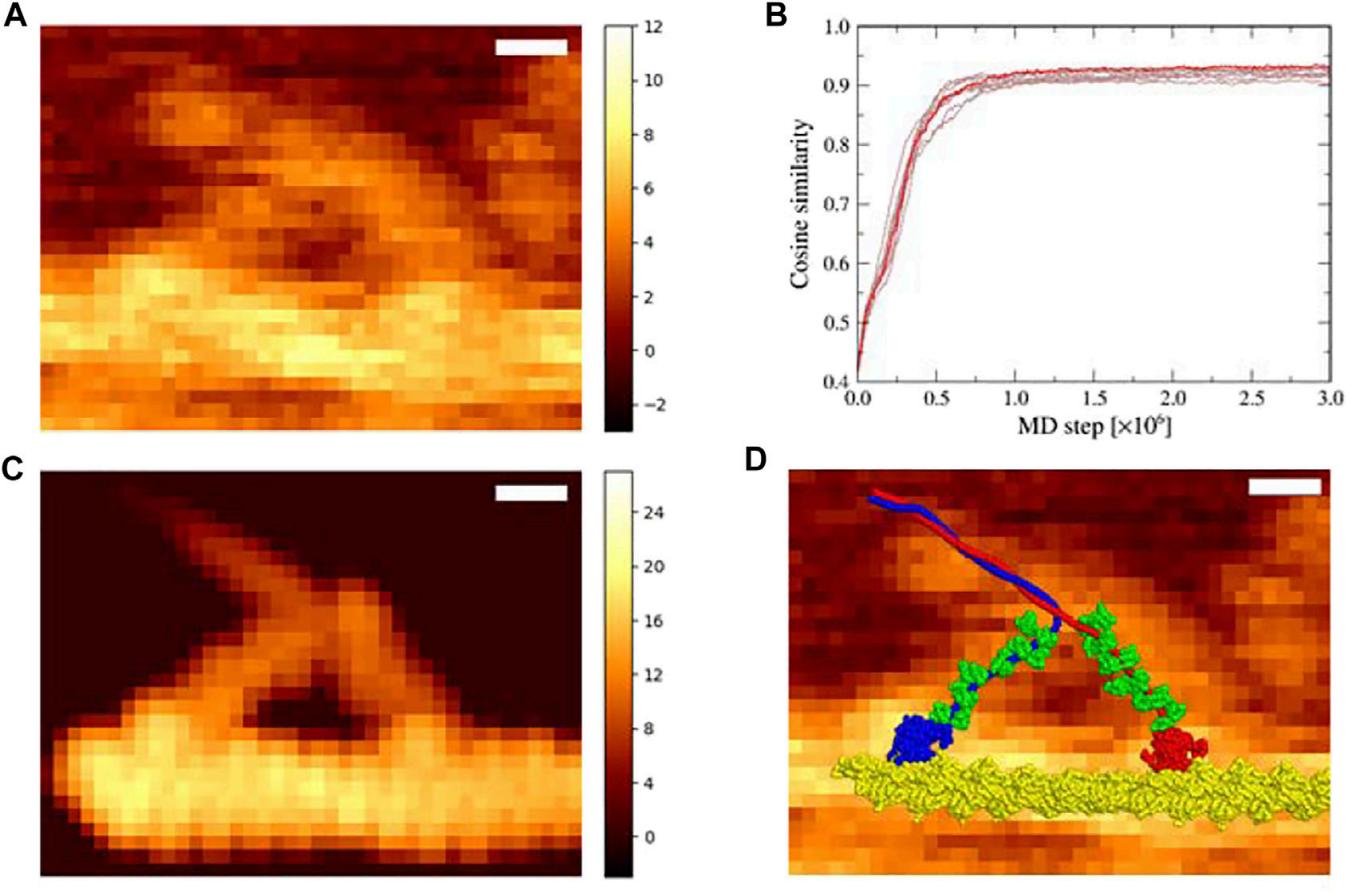
Flexible fitting simulation of the actin-myosin complex in the down-up state. **(A)** The corrected experimental AFM image used as a reference in the simulation; scale bar, 10 nm; color bar in nm. **(B)** Time series of the cosine similarity for ten independent simulation runs are shown in brown. The trajectory involving the largest value is colored in red. **(C)** The pseudo-AFM image with the highest cosine similarity created using afmize (Niina et al., 2020a; Niina et al., 2020b); scale bar, 10 nm; color bar in nm. **(D)** The structure model of actin-myosin complex with the highest cosine similarity on top of the reference AFM image; scale bar, 10 nm.

We examined an HS-AFM movie of myosin V, which moved along the actin filament, obtained by Kodera et al. at 146.7 ms per frame (Kodera et al., 2010). In the movie, myosin V walked along the filament in the right direction. We chose a frame (Figure 2A) in which two myosin heads appeared to be bound to an actin filament, which was immediately before the detachment of the trailing head (the left head in Figure 2A). In fact, in the next frame, the trailing head dissociated from the actin filament. We first transformed the AFM image of the chosen frame such that the stage became parallel to the *XY* plane.

To fit with the transformed AFM image in the region of 40 × 30 pixels (26th–65th pixels in the *X* direction and 1st–30th pixels in the *Y* direction, Figure 2A), we performed flexible-fitting MD simulations of the actin-myosin complex in the down-up state. As an initial structure for the MD simulations, we used a molecular model of the actin-myosin complex in the down-up state generated as described in Section 2.1. This model was considered to be valid and reasonable because each of two myosin heads was in the position to interact properly with the actin filament, where the total value of restraint potentials was sufficiently small, and no significant conformational changes were observed in each of two motor domains of heavy chains (residues 1–764) compared with the homology modeled structure; root-mean-square displacements of the motor domain except for flexible regions (residues 1–4, 53–58, 185–190, and 594–631) were 0.28 and 0.19 Å for trailing and leading heads, respectively. The initial structure was roughly placed by eye so that the entire structure overlapped its AFM image with the trailing head on the left and the leading head on the right. The same simulations were repeated ten times with different random numbers in the Langevin dynamics. In all the simulations, we found that the cosine similarity of pseudo-AFM images increased rapidly and steadily, reaching a constant value above 0.9 (Figure 2B). These results indicate that the flexible fittings to the reference AFM image were performed successfully, and that well-fitted structures were obtained.

The pseudo-AFM image with the highest cosine similarity of 0.936 obtained *via* the simulation (Figure 2C) resembled the reference AFM image, and the corresponding structure of the actin-myosin complex was well fitted to the reference AFM image (Figure 2D). In particular, a characteristic image pattern originating from the double-stranded helical shape of the actin filament was observed in the experimental AFM image, which was well reproduced in the pseudo-AFM image.

Noticeably, the leading neck of myosin V appears to be slightly curved forward in the experimental AFM image but is almost straight in the pseudo-AFM image, making the agreement imperfect in the region.

### 3.2 Flexible Fitting Molecular Dynamics Simulation With the Down-Down State

**FIGURE 3:**
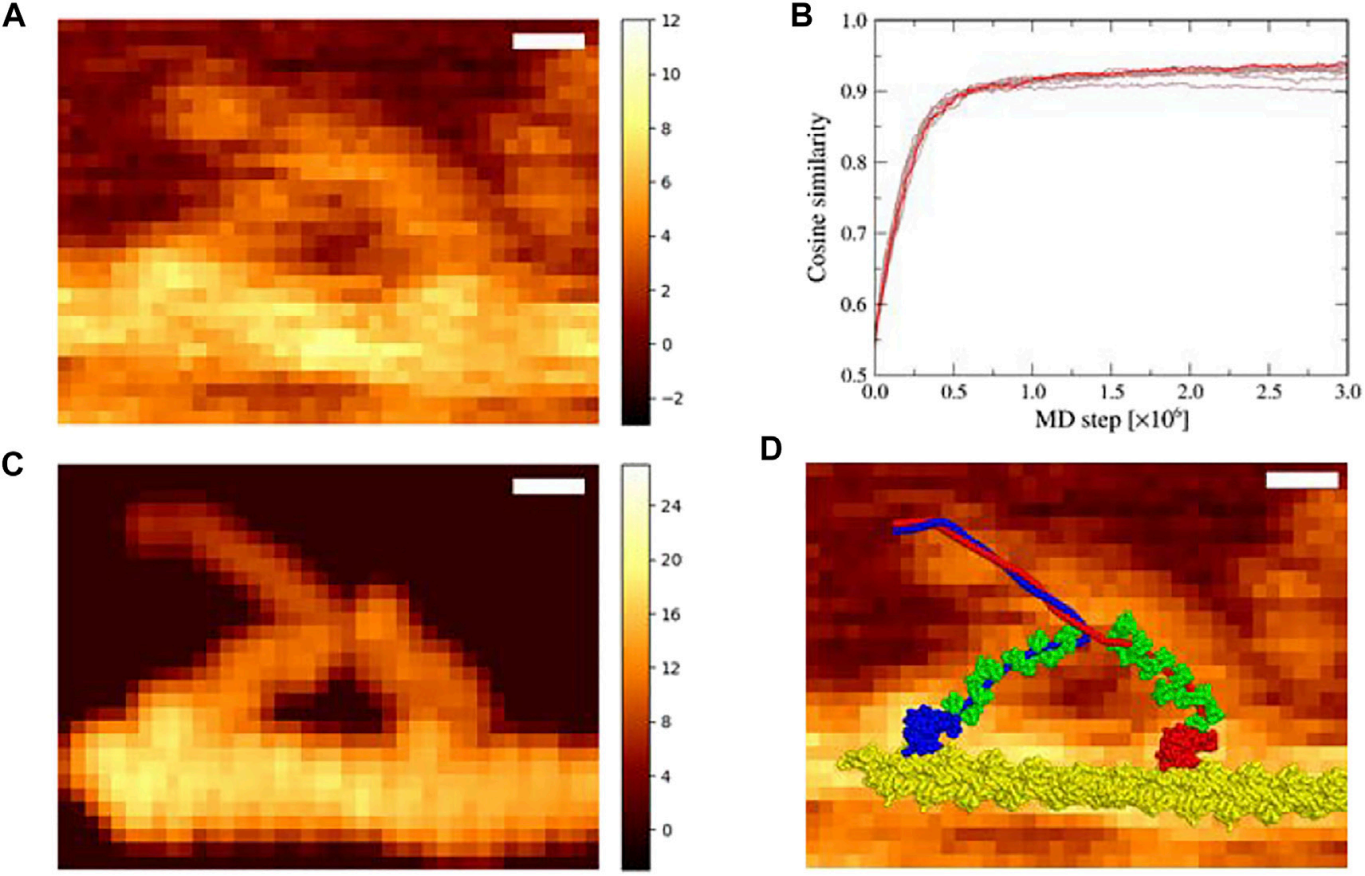
Flexible fitting simulation of the actin-myosin complex in the down-down state. **(A)** The corrected experimental AFM image used as a reference in the simulation; scale bar, 10 nm; color bar in nm (same as Figure 2A). **(B)** Time series of the cosine similarity for ten independent simulation runs are shown in brown. The trajectory involving the largest value is colored in red. **(C)** The pseudo-AFM image with the highest cosine similarity created using afmize (Niina et al., 2020a; Niina et al., 2020b); scale bar, 10 nm; color bar in nm. **(D)** Structure model of the actin-myosin complex with the highest cosine similarity on top of the reference AFM image; scale bar, 10 nm.

Two-head bound state of myosin V is formed from one-head bound state (in which the trailing head in the nucleotide free state is bound in the lever-arm down conformation) by weakly binding of the leading head in the lever-arm up conformation in the ADP–P_i_ bound state (P_i_, inorganic phosphate) to the actin filament (Wulf et al., 2016; Robert-Paganin et al., 2020). Thus, in Section 3.1, using the down-up state of myosin V as the reference structure of the structure-based potential, we performed flexible-fitting MD simulations. However, ADP and P_i_ could be released from the leading head in the down-up state before detachment of the trailing head, especially at a low ATP concentration (1 μM in the experiment for the AFM image used in this study). Upon release of ADP and P_i_, the leading-head lever-arm changes from an up to a down conformation, leading to the down-down state. To examine the possibility that the AFM image represents this state, we performed flexible-fitting MD simulations of the actin-myosin complex with the “down-down” state fitting to the same AFM image (Figure 3A). The initial structure was prepared in the same manner as that in the down-up state.

In ten simulations with different random numbers, the cosine similarity of pseudo-AFM images increased rapidly in the same way as in the case of the down-up state, and saturated over 0.9 value (Figure 3B). These successful results suggest that the reference AFM image could be fitted not only with the down-up state but also with the down-down state. Figure 3C shows a pseudo-AFM image with the highest cosine similarity of 0.941, which is slightly larger than the highest value obtained in the down-up state. The structure of the actin-myosin complex with the highest cosine similarity also fitted well to the reference AFM image (Figure 3D). In the best-fit structure, the leading head of myosin V was in the lever-arm down conformation, which was clearly different from the lever-arm up conformation, and the leading neck of myosin V showed a slightly forward-curved conformation, as expected.

### 3.3 Conformational State Inference of Myosin V in the Atomic Force Microscopy Image

**FIGURE 4:**
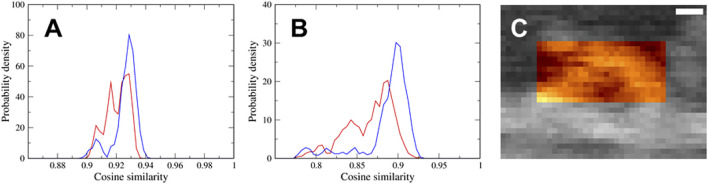
**(A,B)** Probability density distributions of the cosine similarity of pseudo-AFM images using **(A)** the region of 40 × 30 pixels used for the flexible fitting and **(B)** the region of 25 × 12 pixels corresponding to the myosin neck. The distributions of the down-up state and the down-down state are shown in red and blue, respectively. **(C)** The region of AFM image corresponding to the myosin neck is shown in color. Scale bar, 10 nm.

We performed a flexible fitting MD simulation of the actin-myosin complex both with the down-up and down-down states fitting to the same experimental AFM image, and successfully generated well-fitted structures of the actin-myosin complex in both states. To determine which of the two states, the down-up state or down-down state, better represents the myosin observed *via* HS-AFM, we calculated the probability density distributions of the cosine similarity of pseudo-AFM images using the latter half of the time series data of the flexible-fitting MD simulation, that is, 1,500 snapshots. As shown in Figure 4A, the distribution ranges of the two states are almost the same, although the distribution of the down-down state seems to extend slightly toward larger values. This is probably because the region of the actin filament, not the myosin neck, show no significant difference in AFM images between the two states, and contributes significantly to cosine similarity. In fact, there was no obvious difference in the cosine similarity distribution of AFM images for the actin filament region of 40 × 9 pixels (26th–65th pixels in the *X* direction and 3rd–11th pixels in the *Y* direction) (data not shown).

To eliminate the contribution of the actin filament to the cosine similarity and focus on the myosin neck region, we calculated the cosine similarity of the pseudo-AFM images only for the myosin neck region of 25 × 12 pixels (33rd–57th pixels in the *X* direction and 12th–23rd pixels in the *Y* direction, Figure 4C). Results (Figure 4B) showed that the cosine similarity values at the mode were significantly larger in the down-down state than in the down-up state. Thus, we concluded that the down-down state is more probable for the conformational state of myosin observed *via* HS-AFM. This result is reasonable because the reference AFM image used for the flexible fitting was just before the trailing head detachment from the actin filament at low ATP concentrations (1 μM). In a preliminary study, when we performed flexible fitting to another AFM frame image of myosin V that is prior to the current frame by a few frames, we found that the structure with the down-up state fits better than the frame image used in this study.

Each of two cosine similarity distributions shown in Figure 4A appears to consist of three components, and it seems that the positions of each of three peaks in the distribution are mostly identical. To identify the AFM region generating the three components of cosine similarity, we examined AFM images for actin filament region, myosin neck region, and coiled-coil region of 20 × 5 pixels (31st–50th pixels in the *X* direction and 24th–28th pixels in the *Y* direction). In the cosine similarity distributions for actin filament region and myosin neck region, three components were not clearly found, even though myosin neck region may appear to have a few components. On the other hand, coiled-coil domain showed distinctly different conformations, leading to three components in the cosine similarity distribution. However, the variety of conformations in coiled-coil domain was found to be obviously different between the down-up state and down-down state, and the coincidence of the peak positions was considered to be accidental. This conformational variety of coiled-coil domain also affect the myosin neck region of AFM images, resulting in broadening the cosine similarity distributions toward smaller values.

## 4 Discussion

In this study, the position and orientation of the actin-myosin complex were roughly determined using information on the myosin walking direction before performing a flexible-fitting MD simulation. In the case without such information, it is necessary to perform a simulation with the initial structure in various positions and orientations. It would be possible to perform flexible fitting MD simulations with an initial structure in every position and orientation; however, such simulations are undoubtedly expensive and time-consuming. If a molecular structure close to that observed *via* HS-AFM is available, this problem can be solved using a rigid-body fitting method, which allows us to search for an optimal position and orientation thoroughly and exhaustively. Recently, a rigid-body fitting method was developed to simultaneously infer the molecular placement and probe tip shape from an AFM image (Niina et al., 2021). This method was applied to two HS-AFM images—an actin filament and a flagellar protein FlhA_C_.

Experimental AFM images inevitably contain non-negligible noise and artifacts, such as those caused by the detachment of an AFM probe from a molecular surface, called the parachuting effect. In the AFM image of the actin-myosin complex used in the flexible fitting MD simulation, height distribution of pixels in the region considered to be the stage surface was well fitted with a Gaussian distribution with the standard deviation of 0.53 nm, which is a significantly large value. A weak parachuting effect can also be observed in the AFM image, especially in the range corresponding to myosin necks. These noises and artifacts can cause a pixel in the AFM image to have a larger value than the actual value, leading to an inaccurate flexible fitting. In addition, the AFM image used in this study showed not only the actin-myosin complex but also obstacles in the high region at the upper left and upper right of myosin, which were streptavidin molecules placed on the surface of the AFM stage and allowed visualization of the myosin walking process (Kodera et al., 2010). These obstacles can also induce an incorrect flexible fitting. Fortunately, no false flexible fitting was observed in the present results. Otherwise, preprocessing of the AFM image, including noise reduction and removal of problematic pixels, is necessary to solve these problems.

There are several points in the analysis performed in this study that are not in line with an actual experimental situation. First, heights of pixels in two pseudo-AFM images with the highest cosine similarity obtained by flexible fitting MD simulation were overall higher than the experimental results. In the corresponding structure, actin filament was detached from the AFM stage and was floating away even though using the AFM stage potential. This phenomenon seems to be often observed in flexible fitting to an AFM image, but its cause is not yet well understood. Second, an actin filament observed in the AFM image is much longer than the model used in flexible fitting simulation, which length is the minimum required for one-step walking of myosin V. Using a molecular model of a longer actin filament, we could reproduce the AFM image more precisely. Third, we performed the flexible fitting simulation without electrostatic interaction, even though it was known to play an important role in actin-myosin binding, especially interactions between loops (Okazaki et al., 2012). Flexible fitting simulations considering electrostatic interaction are possible, but no significant improvement would be expected due to low resolution of AFM image. Finally, data of all pixels in an AFM image are obtained not at the same time but at different times because heights of pixels are measured sequentially by scanning an AFM probe. This data asynchronicity can lead to a significantly distorted AFM image in the case that the movement of an observed biomolecule is faster than the scanning speed. To overcome this problem, two possible ways using a sequential Bayesian data assimilation approach are available: Kalman smoother method and particle filter method. Recently, it has been reported that distortion and noise in HS-AFM movies could be reduced by applying the Kalman smoother method (Kubo at al., 2020), On the other hand, particle filter method integrating HS-AFM data with MD simulations (Fuchigami et al., 2020) can be extended to apply to asynchronous HF-AFM movies, confirming that this extended method worked well as expected.

## Data Availability

The raw data supporting the conclusion of this article will be made available by the authors, without undue reservation.
